# The Kids Are Alright: Outcome of a Safety Programme for Addressing Childhood Injury in Australia

**DOI:** 10.3390/ejihpe11020039

**Published:** 2021-06-15

**Authors:** Blake Peck, Daniel Terry

**Affiliations:** School of Health, Federation University Australia, 3350 Ballarat, Australia; d.terry@federation.edu.au

**Keywords:** child, injury, prevention, safety, education

## Abstract

Globally, injuries are the leading cause of death and represent the highest burden of ongoing disease amongst children 1–16 years of age. Increasingly, prevention programmes are recognising a growing need for intervention strategies that target children. The purpose of this study was to determine the efficacy of the SeeMore Safety Programme, designed to teach children (4–6 years of age) how to make conscious decisions about their own capabilities related to safety and how to manage risk. This retrospective study examined de-identified pre- and post-programme data from a sample of 1027 4 to 6-year-old pre-school children over the four-year period who participated in the SeeMore Safety Programme. Results show a significant improvement in each of the post-test scores and when compared to the pre-test scores (*p* < 0.001). Children from rural areas, as well as those from areas of greater disadvantage, also showed significant improvement in their pre- and post-test scores (*p* < 0.001). Overall, the findings highlight that the SeeMore Safety Programme over the four-year period demonstrates an increase in the children’s capacity to recognise and identify danger and safety amongst all children, offering great promise for reducing the burden of injury on children, their families and society.

## 1. Introduction

Injury is the leading cause of death and ongoing disease burden globally, and in Australia the picture is no different, with injury being the leading cause of death among those between 1 and 16 years of age [1,2], and the principal cause of hospitalisation in this age group [2,3]. Childhood injury has been described as a major public health concern that is neglected by modern society [4]. Injury often carries long-term disability, resulting in significant impacts for both children and their family [5]. An injured child often experiences ongoing limitations related to their physical abilities, chronic pain and psychological issues [6,7]. Not only is the injured child affected, but their injury can also impact on their family and their community networks [8]. Although childhood injury is costly and life-changing, it is also preventable [9].

The frequency, severity, and potential for life-long disability and death, in conjunction with the significant economic cost associated with them, make childhood injury a leading health problem globally and a major public health issue in Australia. The degree of this burden would suggest that it is reasonable to argue that prevention must be incorporated into child health strategies both nationally and globally [10]. According to the WHO, if high-income countries were to implement programmes using interventions proven to be effective, which considered the special vulnerability of children, more than a thousand children’s lives would be saved each day [11]. The WHO and UNICEF [11], reported that countries such as Sweden began implementing strong injury prevention policies and infrastructure several decades ago, and now have achieved the lowest injury rates in the world. In so doing, these countries have shown that targeted childhood injury prevention programmes can have an impact on reducing the rates of injury [12]. Unsurprisingly, good surveillance data are identified by WHO (2008) as a key factor in the success of the experience in Sweden. Coupled with the recommendations from the WHO and UNICEF [11] to have access to strong injury-surveillance data, this would suggest that there remains some work to do in order to better support the targeting of child injury prevention strategies and to subsequently evaluate their impact.

Further complicating our understanding of the impact of injury prevention, limited research has been directed to what kinds of programmes can make the biggest difference to young children’s capacity to read their environment and act with understanding in relation to their own personal safety. This dearth of research evidence means that the debate continues with regard to the most appropriate age or developmental stage of the child, as well as the most effective pedagogical approach [13]. When considered together, these issues culminate in programmes that risk being inadequately targeted and are unable to justify their success in reducing injury. Consequently, they are not sustainable [13,14].

Injury prevention strategies for children tend to focus on specific injury-risk or safety messages, such as helmet use on bicycles [15] and water safety [16]. Communicating safety and injury-risk messages to children has also been the subject of school-based curricula, but interventions at this level often never undergo rigorous evaluation [17]. A Cochrane review by Orton et al. [13], while acknowledging an increase in the number of injury minimisation programmes delivered at all school levels (pre-school through to tertiary education), concludes that the evaluation of these programmes has suffered from a lack of homogeneity in data collection measures. Interestingly, there was only one study in the review that included children aged 4–5 years of age. This is echoed by a narrative review of published research undertaken by Peck et al. [14], who identified that the studies of childhood injury prevention programmes targeting pre-school children were of poor quality and lacked convincing evidence of effectiveness due to the methods of evaluation. In culmination, there was insufficient evidence of the effectiveness of childhood injury prevention programmes for children attending early learning programmes, requiring further high-quality studies to determine their overall effectiveness and longer-term sustainability.

In contrast to this, and in an Australian context, O’Neill et al. [18] conducted a pilot investigation into a targeted primary injury prevention programme titled ‘SeeMore Safety’ designed for early learning centres (Kindergarten and Pre-School settings) nationally. The pedagogical foundation of the programme recognises that children between the ages of four and six have the ability to make conscious decisions about their own safety and how to manage risk if given the right experiences and knowledge. The programme has been designed for children attending an early learning centre and engages children through an interactive suite of children’s safety books and real-life experiences, as well as the central character, called SeeMore Safety, that encourages children to develop a lifelong ‘safety risk intelligence’ which allows children to become competent risk takers [19]. The programme is ‘modularised’ as 13 books that the early learning centre teacher can choose from to contextualise the safety message as part of the standard early learning curriculum without the need for specialist facilitation, and these books can then be taken home to consolidate the learning sessions, e.g., On the Bike, On the Farm, In the Car, At the Beach, At the Playground, etc. The outcomes of the pilot investigation emphasised that the programme had a positive effect on the children’s behaviour and reasoning about safety [18].

Given the pilot’s nature, O’Neill et al.’s [18] study and the subsequently limited number of early learning centres (n = 7) and children (n = 198) sampled, an independent evaluation of the programme’s outcomes is an important step. In addition, the early learning centres were all situated within the one Local Government Area (LGA), which makes judgement about the programme’s ability to meet the needs of diverse socio-economic groups difficult. Despite this, the SeeMore Safety Programme continues to expand, reaching some 400,000 children across 10,000 early learning centres situated in geographical areas identified as least advantaged and most advantaged in socio-economic terms. Despite this, there is yet to be any large-scale study to investigate if indeed the SeeMore Safety Programme is having an impact on addressing childhood injury in Australia.

Therefore, the aim of the current study is to examine if children (4–6 years of age) attending an early learning centre which has embedded the SeeMore Safety programme within its curriculum can demonstrate an improvement in their pre- and post-knowledge of safety and injury-risk amongst the major injury types that are common to their age bracket across New South Wales and Victoria, Australia.

A series of research questions naturally arise from this aim: (i) is there a difference between in the pre- and post-scores for children engaging in the SeeMore safety programme? and (ii) is there a difference between children from areas of lower socio-economic status when compared to their more socio-economic advantaged peers?

## 2. Methods

The retrospective study examined de-identified pre- and post-SeeMore Safety data from January 2016 to December 2019. The data were obtained from 49 early learning centres located in various towns and cities across Victoria and New South Wales, Australia.

### 2.1. Participants

The SeeMore Safety programme was embedded within the curriculum of participating early learning centres across this period. Parents provided consent for their children to participate in this programme as part of the safety characteristics expected of the early years curriculum. The sample consisted of 1225 four to six-year-old children over the four-year period who, as part of the SeeMore Safety Programme, and embedded within the curriculum of the early learning centre, participated in pre- and post-testing associated with the programme’s implementation at each early learning centre. Among the children, 198 (16.2%) were lost to follow-up due to being absent for post-testing or who had permanently left the early learning centre before the post-tests could be completed, and thus a total of 1027 participants remained. Data such as the sex of the children were not included within the data collection, given the ease of identifiability of the children from a number of the pre-schools.

### 2.2. Instrumentation and Procedure

Programme-level data were collected through a purpose-built pre- and post-test safety quiz response tool. The Safety, Risk, Intelligence (SRI) test first developed by O’Neill in 2006 [13] has been specifically developed to measure children’s safety comprehension. The SRI test designed for children includes picture cards depicting safe and unsafe behaviours [18]. While the tool has not undergone formal statistical validation, it has undergone expert peer evaluation upon its inception. Since then, the SRI has been used for many years across the SeeMore Safety programme and includes a tool with strong face-validity.

Facilitated by a member of the KIDS Foundation, each child is provided with a location free from distraction and is asked to play a ‘safety game’. The child is instructed that they are going to be shown some pictures and asked if they think that SeeMore (the mascot) is ‘safe’ or ‘unsafe’. Once the child understands and is comfortable, the facilitator tells the child that the game is about to start and at this point a timer is started. The child is shown a series of 20 A4 (8-1/4 × 11-3/4 inch) images with SeeMore Safety engaging in an activity associated with the major areas of injury seen amongst children (e.g., SeeMore is at the beach and is swimming outside of the safety flags for water safety). The child is asked to identify if SeeMore is being ‘safe’ or ‘unsafe’. The facilitator adds each answer (correct or incorrect) onto the safety quiz response tool. As soon as the child has given their responses to the various pictures of SeeMore in both ‘safe’ and ‘unsafe’ scenarios, the facilitator stops the timer, and the time is noted on the safety quiz response tool. To reduce the risk of response bias the facilitator shuffles the cards before each testing episode. This process is followed for both the pre- and post-testing phase.

The pre- and post-testing was conducted by the same individual facilitator from the KIDS Foundation one day prior to the commencement of each programme, and then collected six months after the pre-test, which coincides with the conclusion of each programme, throughout the four-year period. The research process outlined here is reflective of the way in which data are collected by the KIDS Foundation in the typical conduct of their programme.

Additional data beyond pre- and post-testing were collected, including the postcode and local government area (LGA) of the early learning centre. These data ensured that each early learning centre was then classified as being located in Metropolitan areas, Regional centres, Large Rural Towns, Medium Rural Towns or Small Rural Towns, as defined within the Modified Monash Model (MMM). The MMM provides a more accurate understanding of Australian rurality by combining both population size and remoteness [20].

Additional data were also obtained from the most recent and publicly available 2016 Australian Census of Population and Housing, specifically the four subsets of the Socio-Economic Indexes for Areas (SEIFA), which include: Index of Relative Socio-Economic Disadvantage (IRSD), Index of Relative Socio-Economic Advantage and Disadvantage (IRSAD), Index of Economic Resources (IER) and Index of Education and Occupation (IEO) [21]. These data were matched to the LGA of the early learning centre to ascertain the socio-economic status of the population within the LGA. It must be noted that without a large enough dataset the various raw scores, deciles and quintiles for each index were classified into a higher level of disadvantage (Decile 1–5) and higher levels of advantage (Decile 6–10).

### 2.3. Data Analysis

Quantitative data were cleaned, checked and analysed using Microsoft Excel and the Statistical Package for the Social Sciences (SPSS, Version 25.0) [22]. Descriptive and interferential statistics were used to analyse data and included an independent sample t-test and one-way ANOVAs. These statistical analyses were used to identify differences according to the metropolitan and rural locations of pre-schools. A preliminary analysis was undertaken to ensure that no violations of assumptions were present. Significance was determined at two-tailed *p* ≤ 0.05 and effect size (d) was determined at 0.01 (small), 0.06 (Moderate), and 0.14 (Large).

## 3. Results

Over the four-year study period, it was indicated that there were 49 pre-schools consisting of 73 groups of children, leading to a total of 1027 children who participated in the research component of the programme. It must be noted that a number of early learning centres participated multiple times in the same year or over the four-year programme (Table 1). 

When examining the mean scores across the 73 groups of children, there was a significant improvement in the pre-test scores when compared to the post-test scores between 2016 to 2019. In addition, the large effect size suggests that the SeeMore Safety Programme has a promising impact on children’s safety awareness. Overall, the findings highlight that the SeeMore Safety Programme over the four-year period demonstrates an increase in the children’s capacity to recognise and identify danger and safety. In addition, it must be noted that children’s response times when identifying safe and unsafe practices within the programme became quicker overtime. However, it must be noted that the way in which response time was measured over the four-year period slightly changed from 2017 onwards, which may account for response times being significantly lower after the children completed the SeeMore safety programme with small effect (Table 2).

Upon further interrogation of each year, it is demonstrated that regardless of the year in which the programme was run, a significant improvement was observed in pre- and post-programme scores among children with a large effect size in each year the programme was run, suggesting that the SeeMore Safety Programme has had an impact on children’s safety awareness each year (Table 3). Overall, regardless of how early learning centre groups had initially scored, all groups showed an increase in scores once completing the SeeMore Safety Programme.

### 3.1. Regional and Metropolitan Differences

Among the 49 early learning centres, the programmes were run at centres located across metropolitan and regional areas of Victoria and New South Wales (MMM1 to MMM5). When examining where children participated in the programme, it was shown that regardless of the geographical location of programme participation, all pre- and post-programme scores demonstrated significant improvements. Regardless of how the children had initially scored prior to undertaking the SeeMore Safety Programme, all groups of children, regardless of location, showed an increase in scores after completing the programme.

It must be noted that children who participated in the programme in Outer Regional settings performed significantly better than their Inner Regional and Major Centre counterparts in pre-testing scores. However, children participating in the programme from Inner Regional areas outperformed children from Major Centres and Outer Regional settings by the end of the SeeMore Safety Programme period. However, it must be noted that the effect size observed in Outer Regional centres was moderate, while Major Centres and Inner Regional centres had large effect sizes. This suggest that the SeeMore Safety Programme has a moderate to high impact on children’s safety awareness in urban, regional, and rural settings (Table 4).

### 3.2. Pre- and Post-Test Differences between Socio-Economic Groups

When examining the socio-economic status according to the postcode of the children, it was noted that regardless of the various measures of socio-economic status, pre- and post-testing scores showed significant improvement, all with large effect sizes. Overall, regardless of how the children had initially scored prior to participating in the SeeMore Safety Programme, all groups, regardless of socio-economic status, showed an increase in scores once completing the programme (Table 5).

### 3.3. Differences between Socio-Economic Groups According to the Type of Test Undertaken

When examining the differences between socio-economic status groups according to the postcode of the early learning centres and the type of test undertaken, it was noted that all pre-test measures were significantly different between high and low SES groups, but with very small or insignificant effect sizes. Nevertheless, it is noted that lower SES groups tended to have lower pre-test scores compared to their higher SES counterparts, except for the measures of IER and IEO.

In addition, when examining post-test scores between high and low SES groups, it was demonstrated that in most cases, all measures were similar regardless of the level of SES, except for the IRSE scores. In this sense, although differences were noted prior to the programme where higher SES groups displayed lower scores, at the end of the programme both high and low SES groups demonstrated similar levels of understanding concerning safety, except for those with higher levels of IRSE.

Overall, children demonstrate differing levels of understanding according to SES levels. However, after undertaking the SeeMore Safety Programme, the gap in understanding becomes negligible regardless of the SES background once the programme is completed (Table 6).

## 4. Discussion

This study is the first large-scale examination of the longstanding KIDS Foundation’s SeeMore Safety Programme and its ability to influence children’s capacity to recognise and identify danger and safety situations. The main outcome of this investigation has been the statistically significant increase in the children’s capacity to read their environment and act with understanding in relation to their own personal safety. Previous research has tended to focus largely on improving parental knowledge and safety practices [23]. While impactful and important in the landscape of injury prevention, injury amongst for children aged 4–6 years tends to occur when children act independently and are not directly supervised. Thus, programmes that focus on improving the children’s understanding of injury risk is essential [17]. Existing programmes have tended to focus on health and safety interventions that take a narrow focus on one injury type, which often carries a requirement for expensive and expert facilitation [13]. The purpose of this study was to evaluate the effectiveness of a programme designed to build a ‘safety consciousness’ applicable in all situations. It must be said that while the SeeMore Safety Programme focuses its attention on the children’s understanding of safety and injury-risk behaviours and not on their actual behaviours in an injury-risk situation, previous research suggests that the former predicts the later [24].

A key element in the development of a safety consciousness is the time that it takes to identify and respond to a safety or injury-risk situation. It follows that if children are able to process their surroundings more quickly and to respond appropriately, they are more likely to avoid injury. The SeeMore Safety Programme was found to decrease the time taken for the children to appraise a practice as safe or unsafe. While the time to appraise and respond to an event has largely been a focus within the discipline of the psychology of learning, it offers an important measure of success of the SeeMore Safety Programme.

Learning and memory can be divided into at least two broad areas of consideration, implicit and explicit, that differ with regard to the way in which what is learned is then accessible [25]. Explicit learning tends to be declarative—the child can verbally articulate what they have learnt, and this knowledge can then be applied in analogous settings. Implicit learning is slower and has been defined as unconscious learning that yields abstract knowledge [26]. The SeeMore Safety Programme certainly provides explicit learning through structured activities but also fosters implicit learning through supported experiences as children engage with risk in their world. While debate continues about the interconnection of explicit and implicit learning, there is a widespread recognition that improvements in reaction times are demonstrative of implicit learning [27]. While we did not specifically seek to measure implicit learning, it would be reasonable to assume that, at some level, the change in the time taken to complete pre- and post-testing is indicative of an unconscious awareness of injury-risk.

It must be stated, at this point, that improvements in knowledge or attitudes towards safety and injury-risk situations, irrespective of the time taken to identify/appraise the risk, should not be conflated with an associated change in behaviour. For both practical and ethical reasons, it is difficult to undertake studies that investigate a child’s ability to avoid injury or risk. Despite this, previous studies have identified an association between injury knowledge and behaviour [24,28], a link requiring further research in the future.

The SeeMore Safety Programme is currently delivered across two large states within Australia (New South Wales and Victoria) that have distinct rural and metropolitan areas as identified using the MMM. This study suggests that children in more regional areas scored better than their urban counterparts with regard to their pre-test scores on safety and injury-risk awareness. Previous research has suggested that children from rural areas tended to have poorer school readiness (or the basic skills that children require to succeed in formal education [29] and tended to lag behind those in other areas [30]. Gan et al. [31] concluded that metropolitan children, in their studies, had a better basic knowledge than rural children and were therefore better prepared for their early learning centre education. The current study suggests, however, that in the context of safety and injury-risk knowledge this is not the case. Worldwide, children in rural areas often have greater exposure to hazards and injury-risk in light of higher levels of agricultural activities [32,33]. Children in non-urban areas are likely to engage with the inherent dangers of: water (dams, rivers), poisons (pesticides), vehicles (tractors, motorbikes) and animal injury (bites) as part of their everyday life. In an effort to mitigate that risk, families often engage in the early education of their children [32], and our findings suggest that perhaps this early exposure to injury and risk might support the higher baseline understanding of safety that we see amongst this cohort.

Another key finding from this study is that the SeeMore Safety Programme is able to transcend the often-challenging SES divide with children from areas considered to be more disadvantaged performing as well in both pre- and post-testing as their more advantaged peers. This is of particular interest in light of other research that suggests that the most socio-economically disadvantaged children lag substantially behind their more advantaged peers with regard to overall Executive Function [34,35], as well as both reading and math skills, which tend to improve along with the socio-economic status [36]. Children from areas of lower SES in the present study were able to perform as well initially and to develop their safety and injury-risk knowledge through the SeeMore Safety Programme to levels at least equivalent to those of their peers from backgrounds of greater advantage. This finding might be explained by the research from Hackman et al. [37], who identified that a child’s level of SES does not predict the rate of growth in their Executive Function.

A study by Morrongiello et al. [17] evaluated a safety awareness programme called ‘Safety Detective’ designed for children aged 4–6 years of age. Of central importance in the programme was its ability to be delivered in a group or classroom approach without the need for expert facilitation and to build safety and injury-risk awareness about injuries that are common around the home, namely burns, falls, poisons or drowning. In addition, in the context of the SeeMore Safety Programme, we see these same requirements reflected where the programme supports children aged 4–6 years regarding the identification of hazards and injury-risk behaviours in the context of injuries that are common amongst the age-group, e.g., falls, being struck by, or collide with, an object. This study has shown that children from different SES backgrounds and from areas with diverse cultural populations can be taught about safety and injury-risk in a group setting from 13 books that the teacher can choose from to contextualise the safety message through the standard early learning centre curriculum without the need for specialist facilitation. Moreover, these books can then be taken home to consolidate the learning sessions, with positive outcomes. Most early learning centres in Australia are mandated to cover safety as part of their curriculum. The SeeMore Safety Programme emerges as a strongly evaluated programme that develops a safety consciousness in children that can be applied in any situation, and importantly, that can be delivered by the teacher at the early learning centre in a child-centred manner. This is achieved by using a central character (SeeMore Safety) to foster a strong connection between the early learning centre and the home as well as to provide a vehicle for at-home learning consolidation. Having an engaging character such as SeeMore Safety helps children to act on the information they receive in a real-world situation [28,38].

## 5. Limitations

For practical reasons, this study did not have a control group to ensure that any change in safety and injury-risk knowledge was not a function of maturation or another confounding variable alone, such as the gender of the facilitator, which may have some influence in the results. In future research, it would be useful to include a control group or groups. Second, although the sex of the children in the study was known, the risk of identifying a particular child or an early learning centre meant that we could not determine the impact of the programme based on this characteristic. Third, the programme focuses on the children’s understanding of safety and injury-risk behaviours and not on their actual behaviours in an injury-risk situation, although previous research suggests that the former predicts the latter and that having an engaging character such as SeeMore Safety helps children to act on the information they receive in a real-world situation. Nonetheless, the capacity for preventative programmes such as this one to impact directly upon the rates of injury amongst the children who have undertaken these programmes is currently missing from the data. Future research that is able to access the links between knowledge and behaviour as well as emergency room presentation data for children experiencing an injury with participation in an injury prevention programme would be valuable. If robust and real-time data on children attending emergency care facilities for injury-based presentation could be accessed, we could link children who had attended a particular programme being run in a particular area by its postcode.

## 6. Conclusions

The SeeMore Safety programme provides an accessible and now evaluated programme that meets an important need within the early childhood research literature. Childhood injury continues to pose a public health concern in need of redress. The structured prevention programmes offered in formal education setting provide a logical point of contact for children and their families. While there is a dearth of research and evidence-based literature to support the programmes that are targeted at younger children, an Australian programme, SeeMore Safety, offers a comprehensive approach to this need. The findings from this study indicate that children (4–6 years of age) attending an early learning centre that has embedded the SeeMore Safety programme within its curriculum demonstrate an improvement in their pre- and post-knowledge of safety and injury-risk amongst the major injury types that are common to their age bracket.

## Figures and Tables

**Table 1:** SeeMore Safety Programme Study Period Data.

Year	Pre-Schools	Number of Groups Run	Total Number of Children
2016	17	17	208
2017	13	13	155
2018	10	11	175
2019	26	32	489
Total	49	73	1027

**Table 2:** SeeMore Safety Programme Pre- and Post-Testing Scores 2016–2019.

Factor	Pre-Test	Post-Test	Difference	t (df)	*p*-Value	d
Response time (seconds)	2.14	1.72	−0.42	3.782 (1828)	*p* < 0.001 **	0.01
Total Score	19.44	22.18	2.74	−22.517 (1825)	*p* < 0.001 **	0.22

** *p* < 0.001.

**Table 3:** SeeMore Safety Programme Pre- and Post-Testing Scores for Each Year (2016 to 2019).

Year	Pre-Test	Post-Test	Difference	t (df)	*p*-Value	d
2016	18.26	21.57	3.31	−3.183 (403)	*p* < 0.001 **	0.02
2017	19.36	22.09	2.73	−8.417 (269)	*p* < 0.001 **	0.21
2018	20.72	22.53	1.81	−6.921 (315)	*p* < 0.001 **	0.13
2019	19.53	22.41	2.88	−16.488 (832)	*p* < 0.001 **	0.25

** *p* < 0.001.

**Table 4:** SeeMore Safety Programme Pre- and Post-Testing Scores 2016–2019 According to Rurality.

Rurality	Pre-Test	Post-Test	Difference	t (df)	*p*-Value	d
MMM1–2 (Major Centre)	19.11	22.06	2.95	−16.471 (881)	*p* < 0.001 **	0.24
MMM3 (Inner Regional)	19.50	22.46	2.96	−14.826 (633)	*p* < 0.001 **	0.26
MMM4–5 (Outer Regional)	20.21	21.89	1.68	−5.968 (308)	*p* < 0.001 **	0.10

** *p* < 0.001.

**Table 5:** SeeMore Safety Programme Pre- and Post-Testing Scores According to Socio-Economic Status.

	SES	Testing Type	Score	Difference	t (df)	*p*-Value	d
IRSD	Low	Pre-Test	19.29	2.79	−17.773 (1091)	0.001 **	0.22
		Post-Test	22.08				
	High	Pre-Test	19.68	2.64	−13.798 (733)	0.001 **	0.21
		Post-Test	22.32				
IRSE	Low	Pre-Test	19.27	2.79	−17.417 (1051)	0.001 **	0.22
		Post-Test	22.06				
	High	Pre-Test	19.68	2.66	−14.248 (773)	0.001 **	0.21
		Post-Test	22.34				
IEO	Low	Pre-Test	19.30	2.69	−15.132 (868)	0.001 **	0.21
		Post-Test	21.99				
	High	Pre-Test	19.58	2.77	−16.657 (956)	0.001 **	0.23
		Post-Test	22.35				
IER	Low	Pre-Test	19.51	2.67	−18.905 (1333)	0.001 **	0.21
		Post-Test	22.18				
	High	Pre-Test	19.26	2.91	−18.905 (1333)	0.001 **	0.21
		Post-Test	22.17				

** *p* < 0.001.

**Table 6:** SeeMore Safety Programme Difference Between Pre- and Post-Testing Scores According to Socio-Economic Status.

	Testing Type	SES	Score	Difference	t (df)	*p*-Value	d
IRSD	Pre-Test	Low	19.29	0.39	−1.986 (1001)	0.047 *	0.00
		High	19.68				
	Post-Test	Low	22.08	0.24	−1.710 (823)	0.088	0.00
		High	22.32				
IRSE	Pre-Test	Low	19.27	0.41	−2.145 (1001)	0.032 *	0.00
		High	19.68				
	Post-Test	Low	22.06	0.28	−1.976 (823)	0.049 *	0.00
		High	22.34				
IER	Pre-Test	Low	19.51	0.25	1.203 (1001)	0.229	0.00
		High	19.26				
	Post-Test	Low	22.18	0.01	0.062 (823)	0.951	0.00
		High	22.17				
IEO	Pre-Test	Low	19.30	0.28	−1.445 (1001)	0.149	0.00
		High	19.58				
	Post-Test	Low	22.18	0.36	0.006 (823)	0.996	0.00
		High	22.17				

* *p* < 0.05.

## Data Availability

Raw data can be made available upon request. The data are not publicly available due to privacy.

## References

[1] NPHP (2004). The National Injury Prevention and Safety Promotion Plan: 2004–2014. National Public Health Partnership.

[2] Australian Institute of Health and Welfare (AIHW) (2020). Australia’s Children.

[3] Australian Institute of Health and Welfare (AIHW) (2011). Headline Indicators for Children’s Health, Development and Wellbeing 2011.

[4] Yanchar N.L., Warda L.J., Fuselli P. (2012). Child and youth injury prevention: A public health approach. Paediatr. Child Health.

[5] Mitchell R., Curtis K., Foster K. (2018). A 10-year review of child injury hospitalisations, health outcomes and treatment costs in Australia. Inj. Prev..

[6] Wallace M., Puryear A., Cannada L.K. (2013). An Evaluation of Posttraumatic Stress Disorder and Parent Stress in Children with Orthopaedic Injuries. J. Orthop. Trauma.

[7] Mehta S., Ameratunga S. (2012). Prevalence of post-traumatic stress disorder among children and adolescents who survive road traffic crashes: A systematic review of the international literature. J. Paediatr. Child Health.

[8] Foster K., Young A., Mitchell R., Van C., Curtis K. (2017). Experiences and needs of parents of critically injured children during the acute hospital phase: A qualitative investigation. Injury.

[9] Mitchell R., Curtis K., Foster K. (2017). A 10-Year Review of the Characteristics and Health Outcomes of Injury-Related Hospitalisations of Children in Australia. Day of Difference Foundation.

[10] Harvey A., Towner E., Peden M., Soori H., Bartolomeos K. (2009). Injury prevention and the attainment of child and adolescent health/Prevention des traumatismes et progres vers la sante de l’enfant et de l’adolescent/La prevencion de las lesiones y la consecucion de la salud del nino y del adolescente. Bull World Health Organ..

[11] Peden M., Oyegbite K., Ozanne-Smith J., Hyder A., Branche A., Rahman A.F., Rivara F., Bartolomeos K. (2008). WHO, UNICEF. World Report on Child Injury Prevention.

[12] Bergman A.B., Rivara F.P. (1991). Sweden’s experience in reducing childhood injuries. Pediatrics.

[13] Orton E., Whitehead J., Mhizha-Murira J., Clarkson M., Watson M.C., Mulvaney C.A., Staniforth J.U.L., Bhuchar M., Kendrick D. (2016). School-based education programmes for the prevention of unintentional injuries in children and young people. Cochrane Database Syst. Rev..

[14] Peck B., Terry D., Ervin K. (2020). A Narrative Synthesis of Childhood Injury Prevention Programs for Pre-School Children. Univers. J. Public Health.

[15] Owen R., Kendrick D., Mulvaney C., Coleman T., Royal S. (2011). Non-legislative interventions for the promotion of cycle helmet wearing by children. Cochrane Database Syst. Rev..

[16] Leavy J.E., Crawford G., Leaversuch F., Nimmo L., McCausland K., Jancey J. (2016). A Review of Drowning Prevention Interventions for Children and Young People in High, Low and Middle Income Countries. J. Community Health.

[17] Morrongiello B.A., Bell M., Park K., Pogrebtsova K. (2016). Evaluation of the Safety Detective Program: A Classroom-Based Intervention to Increase Kindergarten Children’s Understanding of Home Safety Hazards and Injury-Risk Behaviors to Avoid. Prev. Sci..

[18] O’Neill S., Fleer M., Agbenyega J., Ozanne-Smith J., Urlichs M. (2013). A Cultural-Historical Construction of Safety Education Programs for Preschool Children: Findings from SeeMore Safety, the Pilot Study. Australas. J. Early Child..

[19] O’Neill S. (2016). Safety Risk Intelligence: Children’s Concept Formation of Safety and their Individual Capabilities to appraise Risk of Injury. Australas. J. Early Child..

[20] Humphreys J., Wakerman J. (2018). Learning from history: How research evidence can inform policies to improve rural and remote medical workforce distribution. Aust. J. Rural Health.

[21] Australian Bureau of Statistics (ABS), ABS (2016). Socio-Economic Indexes for Areas (SEIFA)—Technical Paper.

[22] IBM Corp (2017). IBM SPSS Statistics for Windows, Version 25.0.

[23] Morrongiello B.A., Zdzieborski D., Sandomierski M., Munroe K. (2013). Results of a randomized controlled trial assessing the efficacy of the Supervising for Home Safety program: Impact on mothers’ supervision practices. Accid. Anal. Prev..

[24] Morrongiello B.A. (2004). Do children’s intentions to risk take relate to actual risk taking?. Inj. Prev..

[25] Thomas K.M., Nelson C.A. (2001). Serial Reaction Time Learning in Preschool- and School-Age Children. J. Exp. Child Psychol..

[26] Reber A.S. (1989). Implicit learning and tacit knowledge. J. Exp. Psychol. Gen..

[27] Sævland W., Norman E. (2016). Studying Different Tasks of Implicit Learning across Multiple Test Sessions Conducted on the Web. Front Psychol..

[28] Claxton L.J., Ponto K.C. (2013). Understanding the properties of interactive televised characters. J. Appl. Dev. Psychol..

[29] Kiernan G., Axford N., Little M., Murphy C., Greene S., Gormley M. (2008). The school readiness of children living in a disadvantaged area in Ireland. J. Early Child. Res..

[30] Stockard J. (2011). Increasing Reading Skills in Rural Areas: An Analysis of Three School Districts. J. Res. Rural Educ..

[31] Gan Y., Meng L., Xie J. (2016). Comparison of School Readiness Between Rural and Urban Chinese Preschool Children. Soc. Behav. Personal. Int. J..

[32] Morrongiello B.A., Zdzieborski D., Stewart J. (2012). Supervision of Children in Agricultural Settings: Implications for Injury Risk and Prevention. J. Agromed..

[33] Lower T., Temperley J. (2018). Farm safety—Time to act. Health Promot. J. Austr..

[34] Morgan P.L., Farkas G., Hillemeier M.M., Pun W.H., Maczuga S. (2019). Kindergarten Children’s Executive Functions Predict Their Second-Grade Academic Achievement and Behavior. Child Dev..

[35] Dickinson E.R., Adelson J.L. (2014). Exploring the Limitations of Measures of Students’ Socioeconomic Status (SES). Pract. Assess. Res. Eval..

[36] Garcia E., Weiss E. (2017). Education Inequalities at the School Starting Gate: Gaps, Trends, and Strategies to Address Them.

[37] Hackman D.A., Gallop R., Evans G.W., Farah M.J. (2015). Socioeconomic status and executive function: Developmental trajectories and mediation. Dev. Sci..

[38] Calvert S.L., Richards M.N., Kent C.C. (2014). Personalized interactive characters for toddlers’ learning of seriation from a video presentation. J. Appl. Dev. Psychol..

